# Using information and communication technologies to involve patients and the public in health education in rural and remote areas: a scoping review

**DOI:** 10.1186/s12913-019-3906-7

**Published:** 2019-02-19

**Authors:** Maman Joyce Dogba, Anara Richi Dossa, Erik Breton, Ruth Gandonou-Migan

**Affiliations:** Department of Family and Emergency Medicine, Faculty of Medicine, Laval University Laval, 1050 Avenue de la Médecine, Quebec City, G1V 0A6 Canada

**Keywords:** Public and patient involvement, Health education, Patient participation, Community engagement, Medical informatics, Continuing professional development, Rural population, Rural areas

## Abstract

**Background:**

Patient and public involvement (PPI) in health education is a practice whereby research and education are carried in collaboration ‘with’ patients and/or citizens, maintaining their role as a team member or expert. PPI in health education is of great interest for all stakeholders in the field, as it can make program development more relevant to the public and increase its utilization by the target population. However, little is known about how PPI should be implemented in different settings particularly in rural and remote areas. Therefore, a deeper understanding of how PPI works in different environments is needed. We aim to explore how information and communication technologies (ICT) are used for PPI in health education programs in rural and remote areas.

**Methods:**

We performed a scoping review. Two reviewers independently selected 641 studies from five electronic databases. Data were extracted, charted and validated by the senior researcher and study lead. We performed a narrative synthesis to map the literature.

**Results:**

Of the initial 641 articles identified, 5 fit the eligibility criteria. Most of the studies targeted community members at large. Consultation and collaboration were the main levels of PPI, which included communities at large and specific at-risk groups. The main forms of ICT used were telephone and Internet, followed by teleconferences, electronic health records, and weblogs. No study measured the effectiveness of ICT for involving patients in health education in rural and remote areas.

**Conclusion:**

Telephone and Internet were the most frequently used forms of PPI in health education in rural areas with consultation and collaboration as the main levels of PPI there. No study measured the impact of ICT for PPI in health education in rural areas. Due to this, measuring the impact of ICT in rural and remote areas as a means for PPI in health education of medical students, health professionals and patients requires further study.

**Electronic supplementary material:**

The online version of this article (10.1186/s12913-019-3906-7) contains supplementary material, which is available to authorized users.

## Background

Patient and public involvement (PPI) refers to a ‘project’ carried out ‘with’ or ‘by’ patients and members of the public rather than them remaining solely subjects and/or ‘recipients’ [1]. To this end, PPI can be applied in research, education or policy development, whereby, patients and/or citizens are included as consultants, advisors and contributing team members. PPI in health education is increasing as there is a growing interest in involving patients and the public as health educators [2–4]. The inclusion of patients and the public, is a collaborative methodology considered essential for improving the relevance of health education across the training continuum for health professionals. It can also be integrated in undergraduate, postgraduate and practical education, continuing professional development (CPD) and in-service training [2, 5]; and is increasingly part of patient education programs [5, 6].

PPI in health education occurs at three levels: consultation, collaboration and partnership [2, 7]. Consultation occurs when patients and the public share their stories or experiences with learners [2] or other patients [7], and may be used to create tools, such as education materials [2] and patient decision aids [7]. Collaboration occurs with active patient involvement, in areas such as, teaching [2], assessment [2], curriculum development [2], or by giving their views and preferences about education priorities [7]. Finally, partnership happens when patients or citizens are involved in decision making at the institutional level. This can include student selection [2], reviewing grant applications [2], sitting on advisory boards or curriculum committees [2], and allocating resources to patient education programs [7].

Towle’s study [2] concludes there “is some evidence of benefits of PPI to students, patients, teachers and communities.” However, it suggests that research should be conducted to understand how PPI works in these specific environments [2]. People from rural and remote areas could be particularly at risk of being excluded from PPI [8, 9] due to the limited availability of services and health professionals [10], distance from healthcare service points [10], and poverty [10]. According to the World Bank, in 2014, 47% of the world’s total population was living in rural areas [11]. It is, therefore, important to improve the use of PPI in rural and remote areas.

Information and communication technologies (ICT) have great potential for improving health education in rural areas [12]. ICT includes all types of telecommunication and broadcasting systems, telecommunication services (landline, wireless, mobile, satellite), computer hardware, software, networks and services, content producing and managing multimedia systems, Internet technologies, and mobile phone applications [13]. Most countries are investing heavily in technology-delivered learning approaches to match the evolving learning styles of medical students [14, 15] and patients with chronic diseases [16–18]. ICT are also used to provide continued professional development for health professionals in rural and remote areas, as lack of it outside of urban centres is a known obstacle to maintaining physicians in these areas [12]. Health education, both rural and urban, is also known to be an area where the needs and views of patients and the public could not be adequately considered [8, 9, 19, 20]. We were interested in the potential utilization of ICT for involving patients and the public in health education specifically in rural areas. We sought to explore two questions: 1) What ICT are currently being used to facilitate PPI in rural health education?; and 2) How effective is the use of ICT for PPI in health education in these areas?

## Methods

In 2015, we conducted a scoping literature review [21, 22]. This methodology synthesizes knowledge that explores a research question by mapping key concepts underlying the domain or research area, main sources and types of evidence available, and the gaps in research [22]. Scoping reviews involve research questions that are broad in nature and require a deep cover of all relevant domains [22]. Contrary to a systematic review, it allows for broad mapping of relevant literature without restricting the review to specific populations, very narrow research questions, certain types of study designs, or high-level academic articles. We sought to answer these two research questions: 1) What ICT are currently being used to facilitate PPI in health education in rural areas?; and 2) How effective is the use of ICT for PPI in health education in rural areas? As our research questions involved the four central interlinked concepts (ICT, PPI, health education, and rural and remote areas), we anticipated a wide range of quantitative and qualitative study designs. As a result, we applied the five steps of the methodological framework proposed by Arskey & O’Malley [22] and Levac et al. [21].

The researchers used a systematic literature search strategy to enhance the methodological quality of the scoping review [21] and identified five electronic databases (PUBMED, EMBASE, Web of Science, CINAHL and ERIC). A research assistant compiled relevant articles which were also validated by a medical librarian. Passive involvement studies were excluded from this study. Two reviewers (ARD and RGM) independently screened studies according to the eligibility criteria. Ambiguous articles were read in full by two reviewers. In cases of divergent opinions on article inclusion, consensus was reached by discussions among the research team members. Expert opinions, editorials and articles that omitted authors’ names or abstracts were excluded.

The eligibility criteria for this study were: 1) research articles published in English or French; 2) that included all four key concepts: i. (PPI [consultation, collaboration or partnership], ii. health education [patient education, medical education and CPD], iii. Rural and remote areas, iv. ICT. We excluded articles that did not specifically include all four concepts. Senior researchers (MJD and EB) distinguished two categories of studies: those that described the ICT without evaluation, and those presenting the outcomes of ICT. The search strategy is outlined in Additional file 1 which can be found in the online appendices supplementary to this manuscript.

ARD and EB extracted and compiled the information contained in the articles using a collection grid developed within the team to summarize key concepts. We completed a pre-test of the grid on a sample of the articles and modified as needed. The collection form included the following items: first author’s name, publication date, country, rural area studied, type and target of health education, type of ICT, study objective, rationale for PPI, level of PPI (consultation, collaboration or partnership), study design, recruitment method, impact measures, results related to PPI, and discussion points about PPI. It was then possible to search for the conditions that can promote or inhibit the use of ICT for PPI in health education in rural areas. We additionally collected outcome data on ICT effectiveness, ICT cost and policy implications.

The team performed a mixed inductive and deductive thematic analysis [23] of the corpus. Data analysis aimed to map three variables: 1) the characteristics of the studies in which these PPI activities took place; 2) the mechanisms used (ICT types); and 3) the observed outcomes, when available. MJD and EB developed a coding dictionary to ensure coding consistency [1]. MJD oversaw the data collection and analysis by ARD and EB.

## Results

### Characteristics of selected studies

**Fig. 1:**
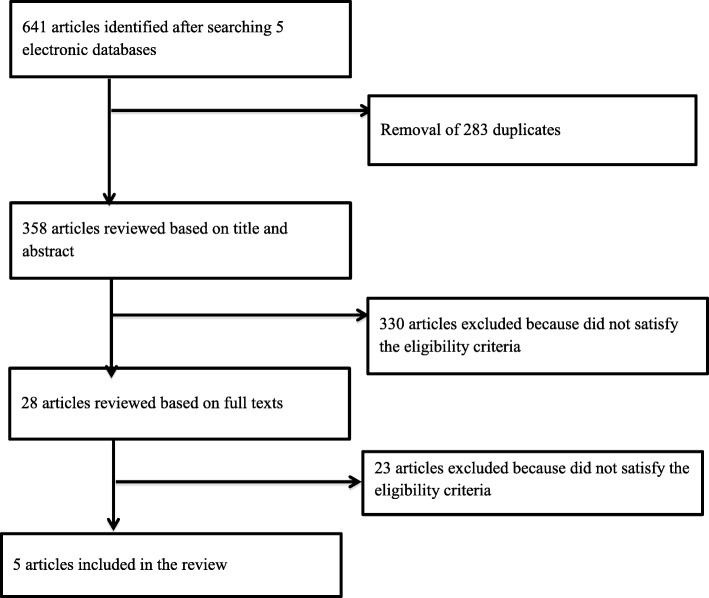
Study Selection Procedure

We found 641 initial articles through the electronic database search. After applying the eligibility and exclusion criteria, we retained 5 articles to comprise the body of this review (Fig. 1). All five were published after 1996 [24–28], with all but one [24–26, 28] being published between 2008 and 2013 (Table 1). Three studies were conducted in the United States (US) [25, 26, 28] and two in Australia [24, 27]. Article study designs included: one review which included a case study [24], one program description [28], and three qualitative survey studies [25–27] (Table 1).

**Table 1:** Characteristics of selected studies

Reference	Author	Year	Country	Design	Objective
[28]	Benson	2013	USA^a^	Program description	To describe the development and implementation of the HeartBeat Connections (HBC)^b^ program as part of a population-based demonstration project aimed at reducing myocardial infarctions. Presentation of a plan of process evaluation focusing on the success of enrolling, engaging, and retaining participants.
[27]	Hays	1996	Australia	Stakeholder consultation	To describe the approach taken at the University of Queensland to broaden the scope of curriculum design to involve rural general practitioners, medical students and rural health care consumers.
[26]	Ramsay	2012	USA	Qualitative: informal needs assessment	To identify the felt needs, desired attributes and acceptability of a stress and depression prevention program for caregivers in four rural areas of Georgia.
[25]	Rogers	2009	USA	Counseling and programming preference study	To determine for rural breast cancer survivors: 1) exercise preference “patterns”; 2) exercise resources and associated factors; and 3) exercise environment.
[24]	Street	2008	Australia	Review and case study	To examine whether it is possible to use the literature both formal and informal to gauge community perspectives on a health technology.

USA^a^: United States of America

HBC^b^: HeartBeat Connections

### Description of study populations and ICT types and application

Table 2 shows the wide variance across studies including, the types of health education utilizing PPI, and the ICT type with its application. Rural areas were located in Minnesota, Georgia and Illinois in the US [25, 26, 28], and in Queensland, Australia [27]. One study did not specify a rural area but sought data on rural and Indigenous populations in Australia [24]. Two studies concerned prevention programs targeting specific at-risk groups [25, 26], two studies involved larger-scale community education programs [24, 28], and one study focused on designing curriculum for medical students [27]. The various specific groups targeted included breast cancer survivors [25] and caregivers [26], while three studies (*n* = 3) targeted the community at large [24, 27, 28]. The most frequent ICT used were the telephone (*n* = 3) [25, 26, 28] and the Internet (*n* = 3) [24–26]. Teleconferencing [27], electronic health records [28], and weblogs [24] were also used. ICT were used for identifying residents at risk, enrolling patients in coaching programs [28], consulting stakeholders about curriculum content [27], as a source of user views on health technology acceptability [24], an itemized ICT preference questionnaire for program dissemination [26] and ICT equipment available in the home that could deliver an exercise program [25].

**Table 2:** Description of study populations and types and function of ICT

Reference	Rural areas	Type of health education	Targeted group	Type of ICT^a^	Function of ICT
[28]	Minnesota (USA)^b^	Coaching on lifestyle behavior change	Residents (*n* = 9) and community representatives (*n* = 36): physicians, local employers, the City of New Ulm Chamber of Commerce, churches, the school district, local colleges, the Brown County Public Health Department, and the community at large	Electronic health records, telephone	Identify residents at risk; enroll patients in coaching program
[27]	Queensland (Australia)	Medical education	Rural community representatives (*n* = 8): Country Women’s Association, the United Graziers’ Association and the National Farmers’ Federation.	Teleconferencing, telephone	Consult stakeholders about new rural curriculum
[26]	Georgia (USA)	Prevention of depression	Participants (*n* = 77): Caregivers = 62 Other groups = 15	Telephone, Internet	Content of survey question about ICT preferences for dissemination of program
[25]	Illinois (USA)	Designing exercise programs	Breast cancer survivors (*n* = 476)	Telephone, Internet	Content of survey question about ICT equipment at home
[24]	Not specified	Health technology assessment (HTA)^c^	Rural, remote and Aboriginal populations	Internet web pages Webblogs	Source of user views on a acceptability of a health technology (reviewed)

ICT^a^: Information and Communication Technologies

USA^b^: United States of America

HTA^c^: Health technology assessment

### Study outcomes regarding ICT use for PPI

No study reported on the effectiveness of ICT for PPI in health education in rural and remote areas, nor on the cost of ICT as a tool for PPI in health education in rural and remote areas. Additionally, no study reported on the policy implications of ICT use for PPI in health education in these areas.

Even if there was no formal evaluation of ICT use for PPI, it was possible to extract the mechanisms. The level of PPI varied across studies, with consultation used in all five studies (*n* = 5) [24–28] (Table 3), and collaboration involved in one [28]. In terms of study methods, two studies used questionnaires [25, 27], two used focus groups [26, 28] and one performed virtual community consultation [24]. The rationale for PPI was explicitly stated in most studies. For two studies, the rationale was to obtain community commitment and engagement with an intervention [24, 28], and in two others it was used to solicit the targeted groups’ views and needs [26, 27]. One study did not report its rationale for PPI [25]. ICT was directly used for carrying out PPI in three studies [26–28] and in two, it was a survey topic [25, 26]. In the studies where PPI was at the level of both consultation and collaboration, focus groups used either telephone or Internet [28]. In the three studies where PPI only used consultation, a questionnaire was administered by teleconference in one study [27], by mail in another [25], and a third reported using weblogs as a form of virtual community [24].

**Table 3:** Mechanisms of Public and Patient and Involvement (PPI)

Reference	Public and patient involvement (PPI^a^)			
	Level of PPI	Method	Rationale for PPI	ICT^b^ used for PPI?
[28]	Consultation	Focus group	The overarching vision of the HONU Project is to create a sustained culture of health in New Ulm, with programs and initiatives for successful replication in other rural communities. The commitment and engagement of the entire community drive the success of all the project’s activities.	Yes
	Collaboration	Steering committee		
[27]	Consultation	Questionnaire	The questionnaire sought their views on the value and acceptability of placing medical students in their communities and also sought their suggestions for making the placements more enjoyable for students.	Yes. (Telephone conference).
[26]	Consultation	Focus group	To design effective interventions for rural caregivers, it is important to consider the unique needs of this population. To design an intervention for likely use, one should consider several aspects of the prior conditions, including felt needs or perceived problems, the degree of innovation and the norms of the social system.	No (survey item) Internet is expensive and impersonal, but could be used to augment programs. Telephone is preferred over Internet, but could be also impersonal and stressful. Phone networks may act as a supplement to support group.
[25]	Consultation	Self-administered mail survey	Not reported	No (survey item) Internet access is limited in rural areas and is not the preferred tool. Because of the ubiquitous nature of telephone access, it is unfortunate that rural survivors prefer telephone delivery.
[24]	Consultation	Weblogs (virtual community consultation)	Community perspectives are important for a number of reasons: to evaluate the acceptability, social impact and potential uptake of a technology; to expand our understanding of the ethical significance of an intervention	Indirectly. Alternative source of information for review with a broader focus on community perspectives. Cannot replace targeted community consultation.

PPI^a^: Patient and Public Involvement

ICT^b^: Information and Communication Technologies

## Discussion

We sought to answer two questions with this scoping review: 1) What ICT are currently being used to facilitate PPI in health education in rural areas?; and 2) How effective is the use of ICT for PPI in health education in these areas? We found that there is a shortage of studies that use ICT for PPI in rural health education, and none that assess its effectiveness. In the few studies found, telephone and Internet were the most used ICT for PPI in rural health education. There is, thus, a serious gap in the literature about the use of ICT for PPI in health education in rural and remote areas. We found no data on its effectiveness and were, therefore, unable to answer our second research question regarding its efficacy.

Due to the paucity and heterogeneity of the studies, it is difficult to generalize on the impact of ICT as a means for PPI in rural health education. Within the sources used for this review, the function of PPI varied between articles with two using PPI to assess needs or preferences related to health programs [25, 26], one used a PPI program description [28], another described a PPI approach to medical education [27], and one used PPI to perform an ICT health technology assessment [24]. Moreover, the function of ICT in PPI was different in each study.

These studies were notable for: 1) the number of qualitative only cross-sectional studies; and 2) the absence or limited discussion of outcome reporting on PPI through ICT use. It would be interesting to assess the temporal relationship between the use of ICT for PPI and outcomes in terms of effective rural health education, but these are data that cannot be gathered in qualitative, cross-sectional studies. Additionally, randomization would make it possible to detect the most effective ICT, as they could be compared for the same form of PPI in health education in rural areas. This could allow outcomes to be qualitatively and quantitatively measured, reported and tabulated as study results [29].

Although quantifiable outcomes of ICT use for PPI in health education were unreported in all selected studies, four of the five discussed the topic [24–26, 28]. One reported the necessity of incorporating electronic health records and using the telephone to engage communities in defining and addressing their health needs; helping more patients prevent disease [28]. Another reported that phone networks were useful in involving patients and the public in preventing depression among their healthcare providers and consequently can contribute to building guidelines for an effective program [26]. The same study mentioned the limitations of using the Internet, reporting that it is expensive and impersonal, but could be used to augment programs [26]. Two studies mentioned telephone preference over using the Internet, but that it can be impersonal and stressful [25, 26]. Finally, one study reported that although weblogs are an alternative source of information for reviews with a broader focus on community perspectives, they cannot replace in-person community consultation [24]. While none of these constitute measurable results, they do suggest some specific limitations of using ICT in rural areas where networks are expensive, limited or non-existent. They also suggest that the personal/impersonal nature of a specific ICT may be a factor in its effectiveness. These suggestions merit further exploration.

This review contains some methodological limitations. First, we did not evaluate the quality of the articles that we included in the review. However, the purpose of a scoping review is to map the key concepts and to detect gaps in the literature, rather than assess literature quality. Second, we did not consult gray literature or retrieve information from the reference lists of the included articles. This may have allowed us to find more articles related to the research questions. A systematic and rigorous process to select articles from five electronic databases did permit us to find the most relevant information. The strength of this review was our adoption of the Arskey framework, which is recommended for conducting scoping reviews. We addressed a limitation of this framework by performing a systematic iterative team approach to selecting articles specific to our research question [21, 30].

## Conclusion

This scoping review found very few studies that reported on the use of ICT for PPI in health education in rural and remote areas. It found that the most frequently used ICT to facilitate PPI were the telephone and Internet. No study reported evaluation of ICT impact as an outcome and therefore, we were unable to answer our second question regarding ICT effectiveness for PPI in rural health education. Thus, further research is needed to measure the impact of ICT as a means for PPI in health education of medical students, health professionals and patients in rural and remote areas.

## Additional file

Additional file 1: Supplementary data associated with this article can be found online. (DOCX 38 kb)

## Abbreviations

CPD: Continuing Professional Development
HBC: HeartBeat Connections
HTA: Health technology assessment
ICT: Information and Communication Technologies
PPI: Patient and Public Involvement
USA: United States of America
