# Supplementary material for: Using information and communication technologies to involve patients and the public in health education in rural and remote areas: a scoping review
Source: BMC Health Serv Res. 2019 Feb 19;19:128. doi: 10.1186/s12913-019-3906-7 (PMC6381697; doi:10.1186/s12913-019-3906-7)
Supplement: Supplementary file 1 — Supplementary data associated with this article can be found online. (DOCX 38 kb) [file 12913_2019_3906_MOESM1_ESM.docx]

**Additional Files**

- Additional file 1: **Search Strategy**

This file reports the search strategy used to identified articles across the five databases (PUBMED, EMBASE, Web of sciences, CINHAL, ERIC) and the number of articles for each query.

### Search strategy for the major database used: PUBMED

| Search | Query | Results |
| --- | --- | --- |
| #5 | #1 AND #2 AND #3 AND #4 | [60](http://www.ncbi.nlm.nih.gov.acces.bibl.ulaval.ca/pubmed/?cmd=HistorySearch&querykey=14) |
| #4 | Search (((rural population*[MeSH Terms]) OR rural health service*[MeSH Terms])) OR ((((rural area*[Title/Abstract]) OR rural communit*[Title/Abstract]) OR rural population*[Title/Abstract]) OR remote rural area*[Title/Abstract]) | [71519](http://www.ncbi.nlm.nih.gov.acces.bibl.ulaval.ca/pubmed/?cmd=HistorySearch&querykey=13) |
| #3 | Search ((((Consumer participation[MeSH Terms]) OR patient participation[MeSH Terms]) OR Decision Making[MeSH Terms])) OR ((((Patient[Title/Abstract] AND public involvement[Title/Abstract])) OR (patient[Title/Abstract] AND family engagement[Title/Abstract])) OR patient focused care[Title/Abstract]) | [151168](http://www.ncbi.nlm.nih.gov.acces.bibl.ulaval.ca/pubmed/?cmd=HistorySearch&querykey=12) |
| #2 | Search (((((Educational Technology[MeSH Terms]) OR medical informatics[MeSH Terms]) OR information services[MeSH Terms]) OR computer communication networks[MeSH Terms])) OR (((((((((((((((((information and communication technolog*[Title/Abstract]) OR information technolog*[Title/Abstract]) OR communication technolog*[Title/Abstract]) OR health informatic*[Title/Abstract]) OR medical informatic*[Title/Abstract]) OR smart card*[Title/Abstract]) OR patient portal*[Title/Abstract]) OR health care blog*[Title/Abstract]) OR computer technolog*[Title/Abstract]) OR mobile application*[Title/Abstract]) OR online application*[Title/Abstract]) OR electronic medical record*[Title/Abstract]) OR telemedicine[Title/Abstract]) OR SMS[Title/Abstract]) OR reminder[Title/Abstract]) OR *phone[Title/Abstract]) OR telecare[Title/Abstract]) | [1320003](http://www.ncbi.nlm.nih.gov.acces.bibl.ulaval.ca/pubmed/?cmd=HistorySearch&querykey=11) |
| #1 | Search (((((Education[MeSH Terms]) OR learning[MeSH Terms]) OR students[MeSH Terms]) OR teaching[MeSH Terms])) OR (((((((((((((Training Program*[Title/Abstract]) OR Educational Activit*[Title/Abstract]) OR Literacy Program*[Title/Abstract]) OR Workshop*[Title/Abstract]) OR Memory Training*[Title/Abstract]) OR Student*[Title/Abstract]) OR School Enrollment*[Title/Abstract]) OR Pedagog*[Title/Abstract]) OR Educational Personnel[Title/Abstract]) OR Training Techn*[Title/Abstract]) OR Training Activit*[Title/Abstract]) OR Educational technic*[Title/Abstract]) OR Teaching Method*[Title/Abstract]) | [1039802](http://www.ncbi.nlm.nih.gov.acces.bibl.ulaval.ca/pubmed/?cmd=HistorySearch&querykey=10) |

### Search strategy for the major database used: EMBASE

| Search | Query | Results |
| --- | --- | --- |
| #5 | #1 AND #2 AND #3 AND #4 | 41 |
| #4 | 'rural population':de,ab,ti OR 'rural health care':de,ab,ti OR 'rural area':ab,ti OR 'rural community':ab,ti OR 'rural care':ab,ti OR 'remote rural area':ab,ti | 51178 |
| #3 | 'education':de,ab,ti OR 'learning':de,ab,ti OR 'teaching':de,ab,ti OR 'student':de,ab,ti OR 'training':ab,ti OR 'educational activities':ab,ti OR'literacy programs':ab,ti OR 'workshop':ab,ti OR 'memory training':ab,ti OR 'student':ab,ti OR 'school enrollment':ab,ti OR 'pedagogics':ab,ti OR'teacher':ab,ti OR 'training technics':ab,ti OR 'educational technics':ab,ti OR 'teaching':ab,ti | 1524217 |
| #2 | 'consumer participation':de,ab,ti OR 'patient participation':de,ab,ti OR 'decision making':de,ab,ti OR 'public participation':ab,ti OR 'patient engagement':ab,ti OR 'family centered care':ab,ti OR 'patient care':ab,ti | 336387 |
| #1 | 'educational technology':de,ab,ti OR 'medical informatics':de,ab,ti OR 'information services':de,ab,ti OR 'computer network':de,ab,ti OR'information technology':ab,ti OR 'information and communication technology':ab,ti OR 'smart card':ab,ti OR 'patient portal':ab,ti OR 'health care blog' OR 'information centre':ab,ti OR 'computer network':ab,ti OR 'mobile application':ab,ti OR 'online application':ab,ti OR 'electronic medical record':ab,ti OR 'telemedicine':ab,ti OR 'sms':ab,ti OR 'reminder':ab,ti OR 'telephone':ab,ti OR 'cellphone':ab,ti OR 'telecare':ab,ti | 113982 |

### Search strategy for the major database used: Web of Sciences

| Search | Query | Results |
| --- | --- | --- |
| # 5 | #1 AND #2 AND #3 AND #4  *Timespan=All years*  *Search language=Auto* | [532](http://apps.webofknowledge.com.acces.bibl.ulaval.ca/summary.do?product=UA&doc=1&qid=27&SID=2BglWHMe5RtkYTq9pmM&search_mode=CombineSearches&update_back2search_link_param=yes) |
| # 4 | TS=(Education OR learning OR education OR students OR Training Program* OR Educational Activit* OR Literacy Program* OR Workshop* OR Memory Training* OR Student* OR School Enrollment* OR Pedagog* OR Educational Personnel OR Training Techn* OR Training Activit* OR Educational technic* OR Teaching Method*)  *Timespan=All years*  *Search language=Auto* | [Approximately](http://apps.webofknowledge.com.acces.bibl.ulaval.ca/summary.do?product=UA&doc=1&qid=26&SID=2BglWHMe5RtkYTq9pmM&search_mode=AdvancedSearch&update_back2search_link_param=yes)  [4,132,640](http://apps.webofknowledge.com.acces.bibl.ulaval.ca/summary.do?product=UA&doc=1&qid=26&SID=2BglWHMe5RtkYTq9pmM&search_mode=AdvancedSearch&update_back2search_link_param=yes) |
| # 3 | TS= (rural population* OR rural health service* OR rural area* OR rural communit* OR remote rural area*)  *Timespan=All years*  *Search language=Auto* | [Approximately](http://apps.webofknowledge.com.acces.bibl.ulaval.ca/summary.do?product=UA&doc=1&qid=24&SID=2BglWHMe5RtkYTq9pmM&search_mode=AdvancedSearch&update_back2search_link_param=yes)  [293,288](http://apps.webofknowledge.com.acces.bibl.ulaval.ca/summary.do?product=UA&doc=1&qid=24&SID=2BglWHMe5RtkYTq9pmM&search_mode=AdvancedSearch&update_back2search_link_param=yes) |
| # 2 | TS= (Consumer participation OR patient participation OR Decision Making OR Patient and public involvement OR patient and family engagement OR patient focused care)  *Timespan=All years*  *Search language=Auto* | [Approximately](http://apps.webofknowledge.com.acces.bibl.ulaval.ca/summary.do?product=UA&doc=1&qid=22&SID=2BglWHMe5RtkYTq9pmM&search_mode=AdvancedSearch&update_back2search_link_param=yes)  [912,611](http://apps.webofknowledge.com.acces.bibl.ulaval.ca/summary.do?product=UA&doc=1&qid=22&SID=2BglWHMe5RtkYTq9pmM&search_mode=AdvancedSearch&update_back2search_link_param=yes) |
| # 1 | TS=(Educational Technology OR medical informatics OR information services OR computer communication networks OR information communication technolog* OR information technolog* OR communication technolog* OR health informatic* OR medical informatic* OR smart card* OR patient portal* OR health care blog* OR computer technolog* OR mobile application* OR online application* OR electronic medical record* OR telemedicine OR SMS OR reminder* OR *phone OR telecare)  *Timespan=All years*  *Search language=Auto* | [Approximately](http://apps.webofknowledge.com.acces.bibl.ulaval.ca/summary.do?product=UA&doc=1&qid=21&SID=2BglWHMe5RtkYTq9pmM&search_mode=AdvancedSearch&update_back2search_link_param=yes)  [1,386,934](http://apps.webofknowledge.com.acces.bibl.ulaval.ca/summary.do?product=UA&doc=1&qid=21&SID=2BglWHMe5RtkYTq9pmM&search_mode=AdvancedSearch&update_back2search_link_param=yes) |

### Search strategy for the major database used: CINHAL

| Search | Query | Results |
| --- | --- | --- |
| #5 | #1 AND #2 AND #3 AND #4 | 3 |
| #4 | MH ( Education OR Learning OR Students OR Teaching ) OR AB ( Training Program OR Educational Activit* OR Literacy OR Seminars and Workshops OR Memory Training OR Student* OR School Admissions OR Conductive Education OR Educational Personnel OR Training Techni* OR Training Activit* OR Educational technic* OR Teaching Method* ) | 140,916 |
| #3 | MH ( Rural Population OR Rural Health Services ) OR AB ( Rural Areas OR Rural Health OR Rural Health Centers OR Rural Health Nursing OR Rural Population OR Remote Consultation OR remote rural area ) | 23,048 |
| #2 | MH ( Consumer Participation OR Consumer Participation OR Decision Making ) OR AB ( Patient and public involvement OR Patient Advocacy OR patient and family engagement OR Family Involvement OR Patient Centered Care ) | 51,599 |
| #1 | MH ( Educational Technology OR Information Science OR Information Services OR Computer Communication Networks ) OR AB ( information and communication technolog* OR information technolog* OR Communications Media* OR Health Informatic* OR medical informatic* OR smart card* OR Patient Record Systems OR patient portal* OR Blog* OR Computers, Portable OR Wireless Communications OR Online Services OR Computerized Patient Record OR Telemedicine OR Text Messaging OR Reminder System* OR *phone* O [...](javascript:showHistoryTerm('ctl00_ctl00_MainContentArea_MainContentArea_historyControl_HistoryRepeater_ctl04_ellipsis',true)) | 40,084 |

### Search strategy for the major database used: ERIC

| Search | Query | Results |
| --- | --- | --- |
| #5 | #1 AND #2 AND #3 AND #4 | 5 |
| #4 | SU ( Education OR Learning OR Students OR Teaching Methods ) OR AB ( Training Methods OR Educational Counseling , Educational demand , Educational development OR Literacy OR Workshop OR Memory Training OR Teacher Student Relationship OR Enrollment OR Instruction OR Schools of Education OR Training Methods OR Training Activit* OR Educational technic* OR Teaching Methods ) | 522,353 |
| # 3 | MH ( Rural Population OR Rural Health Services ) OR AB ( Rural Areas OR Rural Health OR Rural Health Centers OR Rural Health Nursing OR Rural Population OR Remote Consultation OR remote rural area ) | 23,048 |
| #2 | MH ( Consumer Participation OR Consumer Participation OR Decision Making ) OR AB ( Patient and public involvement OR Patient Advocacy OR patient and family engagement OR Family Involvement OR Patient Centered Care ) | 51,599 |
| #1 | MH ( Educational Technology OR Information Science OR Information Services OR Computer Communication Networks ) OR AB ( information and communication technolog* OR information technolog* OR Communications Media* OR Health Informatic* OR medical informatic* OR smart card* OR Patient Record Systems OR patient portal* OR Blog* OR Computers, Portable OR Wireless Communications OR Online Services OR Computerized Patient Record OR Telemedicine OR Text Messaging OR Reminder System* OR *phone* OR telecare OR telecommunication* | 40,084 |
